# The Use of TADs in the Mandibular Arch to Prevent Proclination of the Lower Incisors during the Use of the Mini Scope Herbst Appliance

**DOI:** 10.1155/2022/9144900

**Published:** 2022-10-12

**Authors:** Domenico Aiello, Angelo Finamore, Andrea Scribante, Michele Mario Figliuzzi, Sergio Paduano

**Affiliations:** ^1^Department of Health, University “Magna Graecia” of Catanzaro, Viale Europa, Loc. Germaneto, Catanzaro 88100, Italy; ^2^Unit of Orthodontics and Paediatric Dentistry, Section of Dentistry, Department of Clinical, Surgical, Diagnostic and Paediatric Sciences, University of Pavia, Pavia 27100, Italy

## Abstract

Class II malocclusions are the most frequent within the Italian population. Normally, these malocclusions are caused by a reduction in a mandibular component whose functional stimulus is still very much cause for debate. The negative effect of all types of Class II functional appliances is in the proclination of the lower incisors, which, in subjects whose incisors are already labially inclined, must be checked at all times to avoid serious consequences to these elements. In this case study, a girl aged 14 years and 5 months presented with Class II malocclusion, 2^nd^ division with a convex profile and a visibly retruded chin. The lower incisors presented a marked proclination (−1/Go-Gn ini = 107.7°) in a brachyfacial patient. To avoid further inclination of the lower incisors a Herbst appliance was mounted in two separate sittings. The first part of the appliance including the tubes was mounted to the upper jaw allowing the vestibularisation of the upper incisors in order to increase the overjet. Once this was obtained the lower part of the appliance was mounted together with the telescopic arms associated with two temporary anchorage devices (TADs) in positions 36–37 and 46–47, and an anterior section 33–43 with distal loop to which two double metallic ligatures were anchored at the TADs to contrast the negative effect of the appliance. At the end of the first functional phase, the treatment was refined using MBK fixed therapy to finish the case. The orthodontic therapy led to a visible improvement of the profile and the achievement of a first-class dental–skeletal result on both sides. From the cephalometric evaluation carried out immediately after the Herbst appliance treatment at time *T*1 and at the end of the orthodontic therapy *T*2 it was possible to verify a slight increase in the inclination of the lower incisors (−1/Go-Gn fin = 108°). In conclusion, it can be said that the use of the skeletal anchorage avoided, in this case, the proclination effect in the lower incisors due to the use of a Herbst appliance.

## 1. Introduction

There can be no doubt that Class II malocclusions are the most frequent type found in the Italian population [1]. Most of these malocclusions are not due to a simple dental problem but have a more skeletal–dental nature, with the mandibular component being smaller than average or at least being set further back than is normal [2]. The skeletal component means that these malocclusions have an important impact at the profilometric level [3–6] which is characterised by a convex development and the patient's nose has a greater visual impact due to the repositioning of the chin. In international literature, however, it is still not clear whether, and how much, growth stimulation is possible. In their approach to this problem, orthodontists are divided into those who use a functional type of approach and those who simply seek to compensate the dentition in order to achieve a stable occlusion that lasts over time [7–10]. This latter approach is adopted by those who believe that they cannot modify the patient's profile using orthodontic mechanics and that in order to improve the profile one must fall back upon orthognathic surgery [7, 11–14]. The functional approach, which is unquestionably the option that the majority of clinicians use, also enjoys divergent opinions concerning the type of approach and the timing of the therapy. However, there can be no doubt that a functional approach during the growth peak will lead to more rapid and predictable results since one is intervening in a phase during which the patient's own body is already predisposed towards important growth. As a result, when using a functional treatment, it is sufficient to direct the mandibular growth vector in order to obtain the best results and the greatest stability over time [8, 15–18]. The Herbst fixed functional appliance presents considerable advantages, chiefly due to the reduction in the collaboration required from the patient. Unlike other mobile appliances or Class II elastics, in fact, it cannot be removed and works 24 hours. Unfortunately, exteriorising the force at the level of the definitive molars leads to a distalising vector that is charged to the upper arch and a mesialising one to the lower arch which then leads to the proclination of the lower incisors, more so than with any other functional appliances [19, 20]. In recent years, in attempts at reducing the negative effects on the patient's teeth, many appliances supported by temporary anchorage devices (TADs) have appeared, especially for those concerning rapid palate expanders [21, 22] including appliances to achieve sagittal corrections, such as the Herbst one [23, 24]. The latter allows a considerable reduction in the proclination of the lower incisors and attempt to provide the greatest skeletal effect possible. A systematic revision carried out by Al-Dboush et al. in 2021 seems, albeit with limited evidence, to scientifically support this line of reasoning [25].

## 2. Case Report

This ‘case report' wishes to show how, with of 2 TADs in the lower arch, it is possible to manage the anterior anchorage of the lower teeth in a patient treated with a Herbst without suffering from proclination of the incisors.

## 3. Diagnosis and Aetiology

The patient, aged 14 years and 5 months, came to our notice at the Department of Ondontostomatology at the “Mater Domini” Hospital of Catanzaro. During anamnesis the patient reported an initial orthodontic treatment that was not completed due to loss of trust in the clinician, there were no important pathologies or negative habits or odontological interventions of significance.

**Figure 1:**
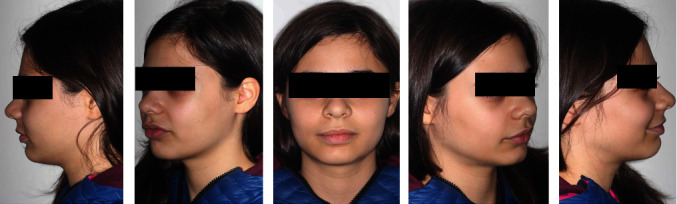
Pre-treatment extraoral photographs.

**Figure 2:**
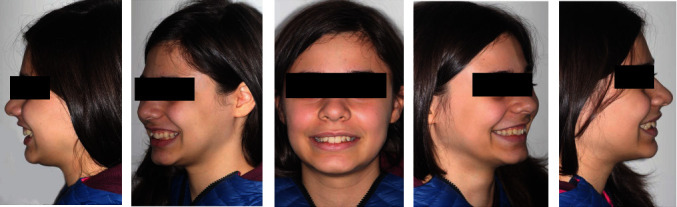
Pre-treatment extraoral photographs of smile.

**Figure 3:**
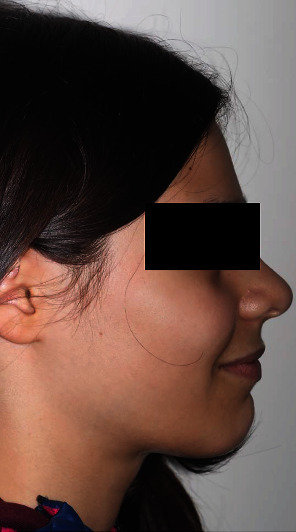
Fränkel manoeuvre.

The extraoral exam (Figures 1 and 2) show a marked convex profile with an important retrusion of the mandible, a concave profile with an augmented labial groove. The Fränkel manoeuvre (Figure 3) show a distinct improvement in the patient's profile. At a functional level, there was no gnathological problems, such as noises or painful articulations.

**Figure 4:**
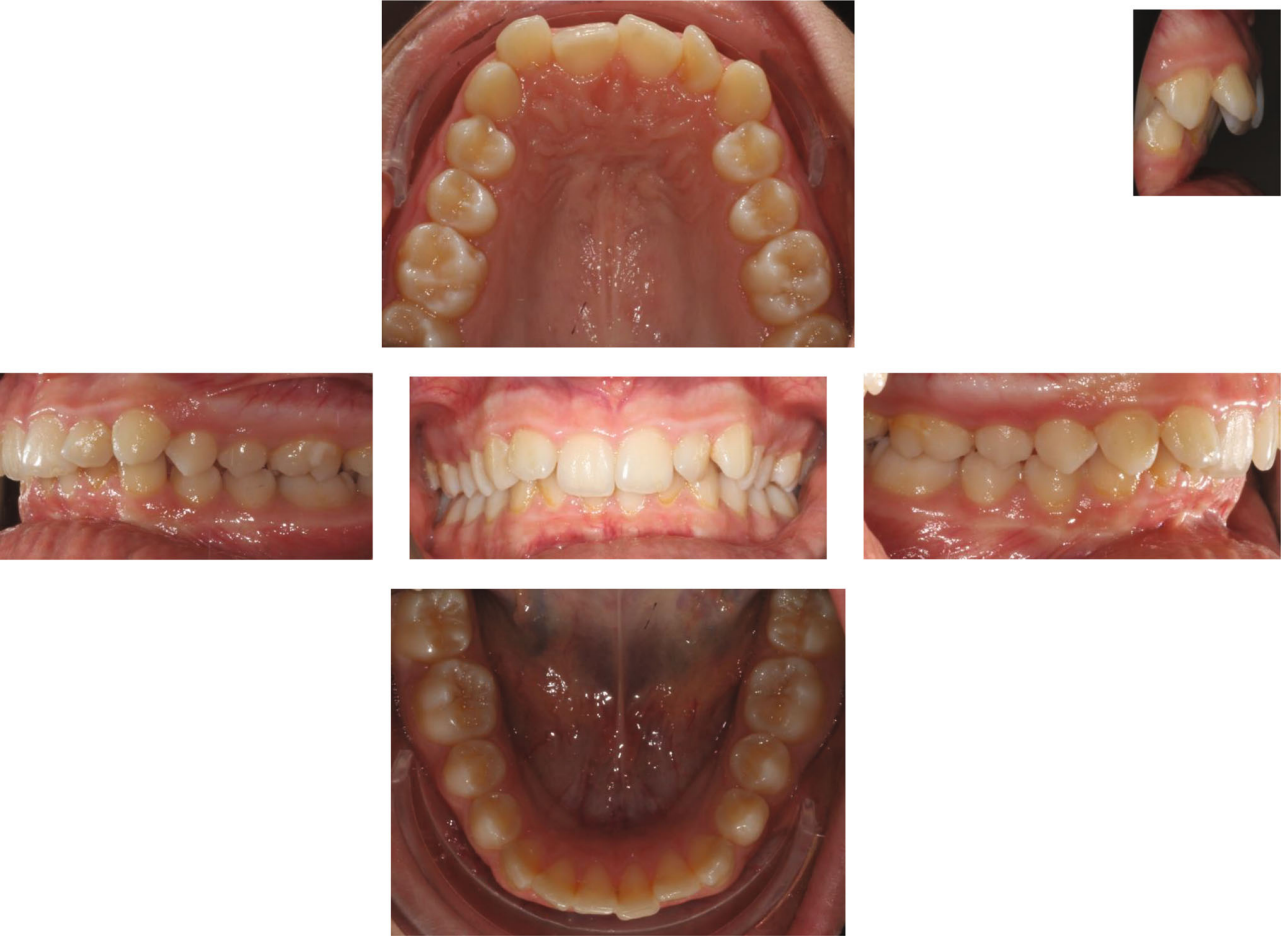
Initial intraoral photographs.

**Figure 5:**
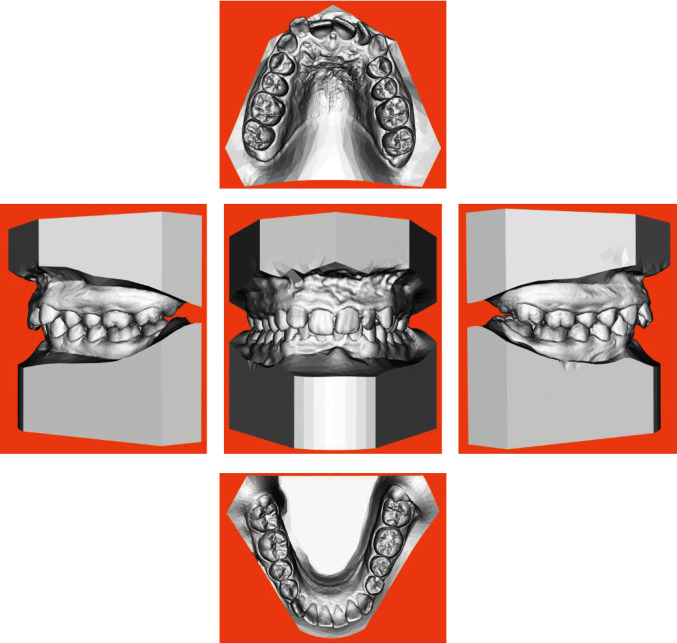
Initial digital model.

The intraoral exam (Figures 4 and 5) show a clear case of Class II, division 2 malocclusion characterised by slightly augmented overjet (OVJ) and overbite. The molar–canine relationship was wholly bilateral Class II and the first upper molars presented a clear mesiopalatal rotation.

**Figure 6:**
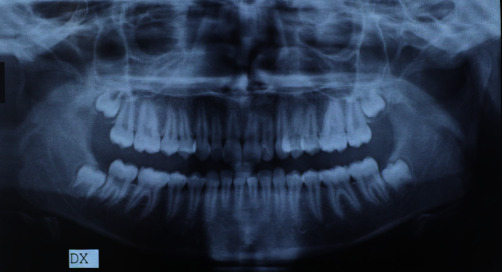
Initial panoramic.

The panoramic X-ray (Figure 6) demonstrated a good oral health condition with the complete eruption of all the dental elements with the exception of the wisdom teeth.

**Figure 7:**
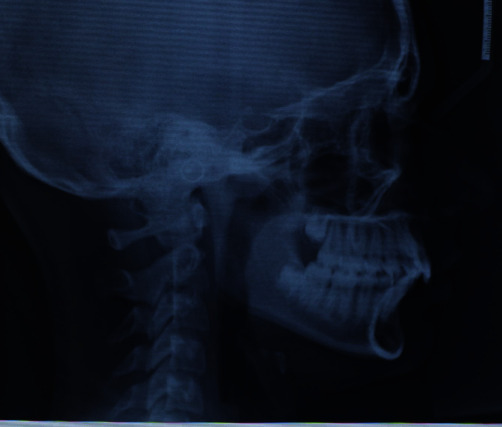
Initial teleradiography.

The cephalometric analysis (Figure 7, Tables 1 and 2) reveal a skeletal Class II characterised by an increase in the AN/Pg angle (6.6°) which in this patient was hypodivergent (SN/Go-Gn = 28° and Ans-Pns/Go-Gn = 18.1°). The upper incisors were shown to be slightly reduced compared with the norm. The lower incisors revealed a noticeable proclination, even before the start of the therapy, of about 6.7° (−1/Go-Gn = 107.7°), whilst the upper ones revealed a slight retroclination (+1/ANS-PNS = 101.1°).

**Figure 8:**
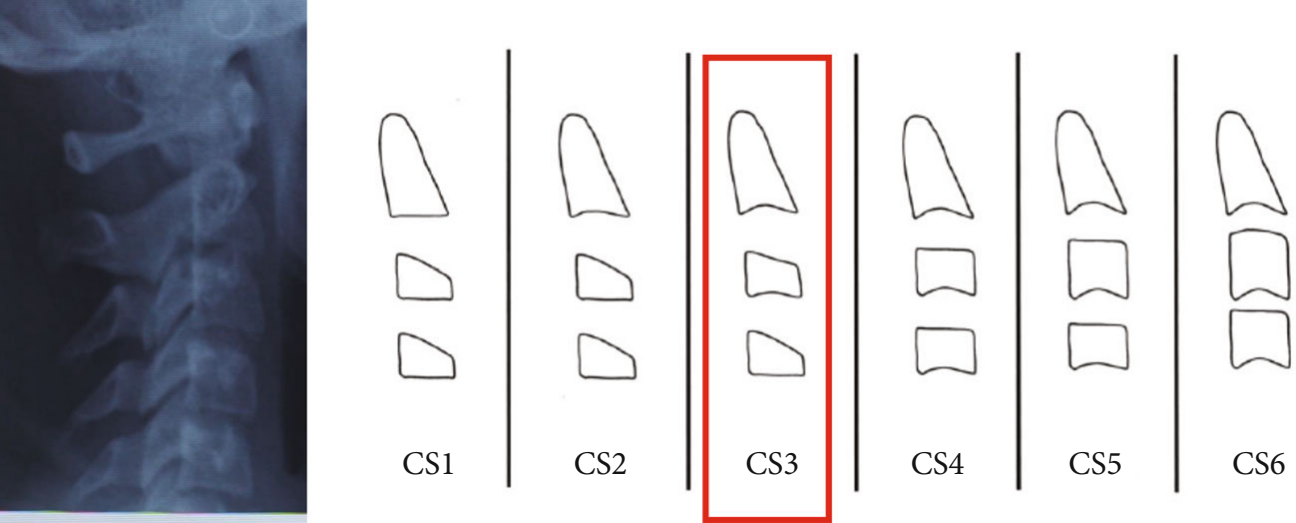
Vertebral stadiation.

According to the analysis of the vertebral staging (Figure 8), the patient can be classified in the type CS3 [26] since the lower concavity is inferior to c2 and c3 but not inferior to c4 and therefore the patient is close to the peak of the growth.

## 4. Therapeutical Objectives

The therapeutical objectives for this patient are as follows: (1) to resolve the upper retroclination in order to consent the advancement of the mandible, (2) to stimulate the growth of the mandible to favour the advancement of the same and thus the resolution of the Class II malocclusion, (3) to correct the mispositioning of the dental elements to consent a correct first-class closure in the molars and canines, (4) to avoid further proclination of the lower incisors, and finally (5) to improve the profile, favouring an augmented prominence of the pogonion of the soft tissues.

## 5. Therapeutic Alternatives

In order to reach the goals we have identified 4 possible therapeutic alternatives.

The first therapy option consist of a fixed therapy, multibracket, and the extraction of the first upper premolars and the compensation of the malocclusion ending with Class I canines and Class II molars. This therapy was discarded immediately since it did not fulfil therapeutic objective 5 and it would have worsened the patient's profile because of its high probable withdrawal of the upper maxilla.

The second therapy option consist of a fixed orthodontic therapy, multibracket, extraction in of the second upper premolars, and the first lower premolars preparation for orthognatical surgery allowing a noticeable improvement in the inclination of the lower incisors and a harmonious effect on the profile through surgery. This option was not up to the patient's liking due to its invasive quality.

The third therapy option consist of a two-phase therapy. The first phase involving a removable device (Sander type) with the lower incisors totally covered in resin in order not to lose the anchorage for about one year. Following this a fixed-device therapy in order to finish off the case and to allow correct intercuspidation.

**Figure 9:**
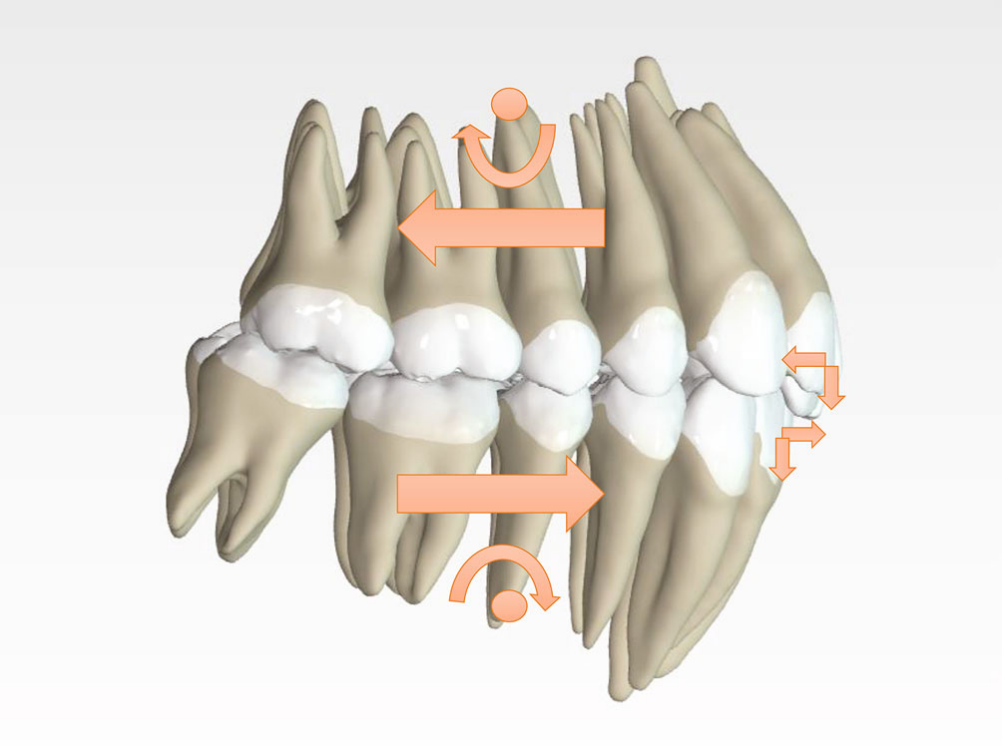
Herbst appliance force.

**Figure 10:**
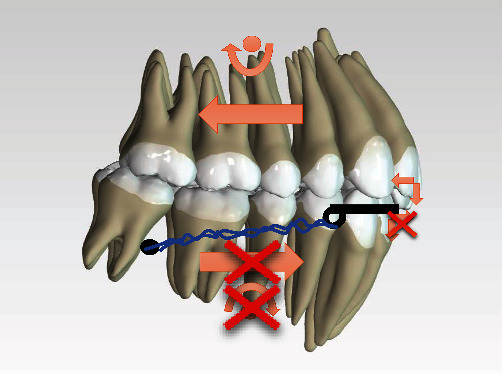
TADs effect on Herbst appliance force.

The fourth therapy option involve the use of a functional, Herbst type fixed-device, mounted in two separate sittings. During the first sitting the application of the upper element with the aim of being able to proclinate the upper incisors using a methodical fixed-device 4 × 2. During the second sitting the application of the lower element together with the telescopic arm to promote mandibular advancement. In order to avoid the proclination of the lower incisors as a result of the use of this device (Figure 9), the association of a systematic skeletal anchorage to contrast the vestibularising force (Figure 10) was used. The whole therapy option has to be finished with a nonextractive, multibracket therapy.

Since the patient was close to her growth peak and permanent dentition, the fourth therapy option was chosen in order to allow the most efficient treatment possible, to reduce the time involved in the therapy, and to promote the greatest possible mandibular advancement.

## 6. Therapeutic Management

Thus, the patient underwent a treatment involving the creation of a Herbst device made up of 4 distinct parts made of fused bands, using the lost-wax method, on the first and second molars. Initially, only the upper bands were cemented which had not only the applecore screws (American Orthodontics, Sheboygan, WI, USA), but also two vestibular tubes. Once the bands were cemented, the 4 upper incisors were bonded using attachments (ovation brackets; Dentsply-Sirona GAC, Bohemia, NY, USA) with Roth prescription extratorque using the orthodontic composite Greengloo (Ormco, Brea, CA, USA). The upper incisors were therefore vestibular-inclined and rotated using a series of arches with Accuform forms:

- 0.016 Ni–Ti (sentalloy; Dentsply-Sirona GAC),
- 0.017 × 0.025 Ni–Ti (sentalloy; Dentsply-Sirona GAC), and
- 0.017 × 0.025 SS (Dentsply-Sirona GAC).

**Figure 11:**
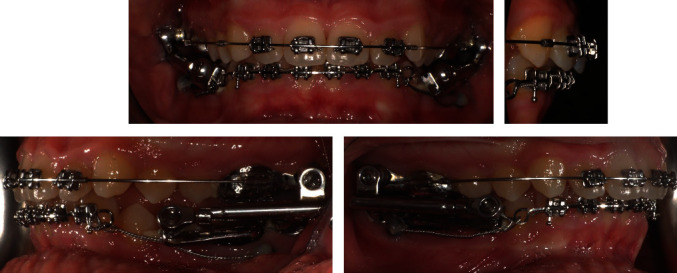
Herbst appliance with TADs.

Once the correct OVJ was achieved (about 10 mm), the two lower bands of the Herbst device were mounted and connected to the upper part with apposite telescopic arms attached to the bands with applecore screws at the top and at the bottom. In the same sitting, a mini-screw was inserted, psm four plus, 1.5 mm × 7 mm (Psm Medical Solution, Gewerbestraße, Gunningen, Germany), on each side [27]. These were inserted interradicularly between the 36 and 37 first and then between the 46 and 47. The elements 31, 32, 33, 41, 42, and 43 were then bonded with the attachments described above and a 0.017 × 0.025 SS sectional with 2 distal helix loops was inserted on the canines. Finally, the screws were linked to the sectional loops using a long, tightly woven, double ligature in order to avoid proclination (Figure 11).

The patient wore the Herbst device for a period of about 12 months and required two reactivations with apposite thicknesses (crimpable shims, American Orthodontics), 1 mm thick after 6 and 8 months from the start of the therapy using the Herbst device. At the end of the functional phase the device was removed together with the mandibular screws. The binding of the residual teeth was completed and bands were inserted on the upper and lower sixes (Dentsply-Sirona GAC). The rotation of the upper sixes was managed using the Gosgharian transpalatal bar with distal loops (Dentsply-Sirona GAC). During this phase another X-ray was carried out, *T*1 time, to allow an assessment of the position of the lower incisors. Finally, a classic sequence of arches was inserted, 0.016 NiTi, 0.017 × 0.025 NiTi, 0.019 × 0.025 NiTi (sentalloy; Dentsply-Sirona GAC), 0.019 × 0.025 SS, and to finish off, some arches, 0.019 × 0.025 SS Multibraid (Dentsply-Sirona GAC) were used to refine the intercuspidation. After the removal of the bands, the patient used a removable containment device for the upper teeth with a thermo-printed, fixed device for the lower teeth using a multibraid 0.195 SS bonded from canine to canine.

## 7. Results of the Treatment

**Figure 12:**
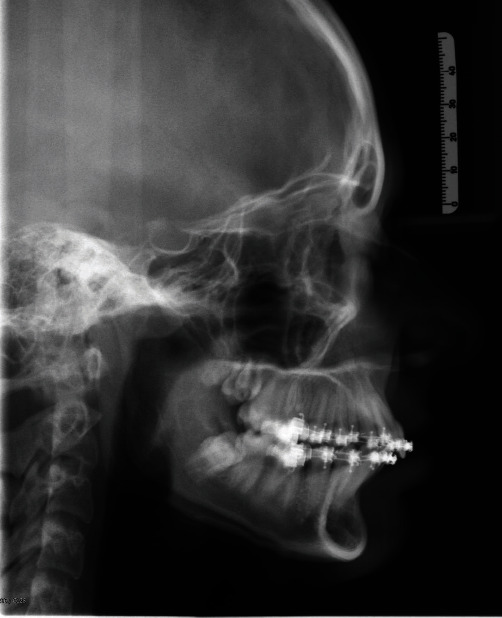
Post-Herbst teleradiography.

After the first phase of therapy with the Herbst device it is possible to note (Figure 12; Tables 3 and 4] how the device promoted mandibular growth increasing the distance Olp-B by more than 2 mm (Olp-B pre/Olp-B post = 2.6 mm) and the Olp-Pg distance by more than 3 mm (Olp-pre/Olp-Pg post = 3.5 mm) according to Pancherz's cephalometry [28]; the labial groove expanded, Olp-Sub (Olp-Sub pre/Olp-Sub post = 4.1 mm) and the chin projection at the profilometric level increased, (Olp-Pgs pre/Olp-Pgs post = 3.8 mm). On the other hand, the skeletal values, Olp/a, Olp/ANS, for the upper mandible remained practically unvaried. Instead, according to Ebo cephalometry, it is possible to note the normalisation of the relationship of Class II to Class I with AN/Pg going from 6.6 to 4.4° whilst at the dental level the angular value of the upper incisors was noticeably increased (+1/ANS-PNS pre/+1/ANS-PNS post = +13.1°) and the value of the lower incisors remained substantially stable (−1/Go-Gn pre/−1/Go-Gn post = 0°), unlike when a Herbst device is used alone without the assistance of mini-screws which leads to an increase of the inclination of the incisors of between 6 and 7.1°, on average [29].

**Figure 13:**
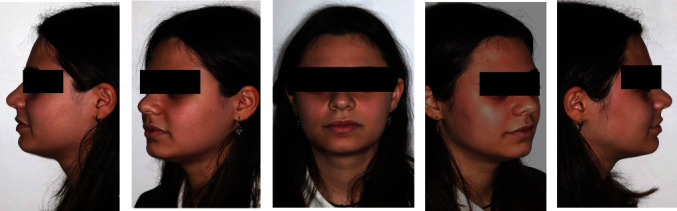
Final extraoral photographs.

**Figure 14:**
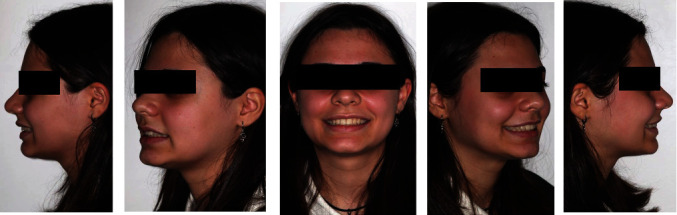
Final extraoral photographs with smile.

**Figure 15:**
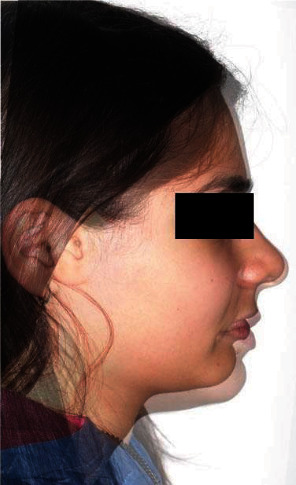
Profile superimposition.

**Figure 16:**
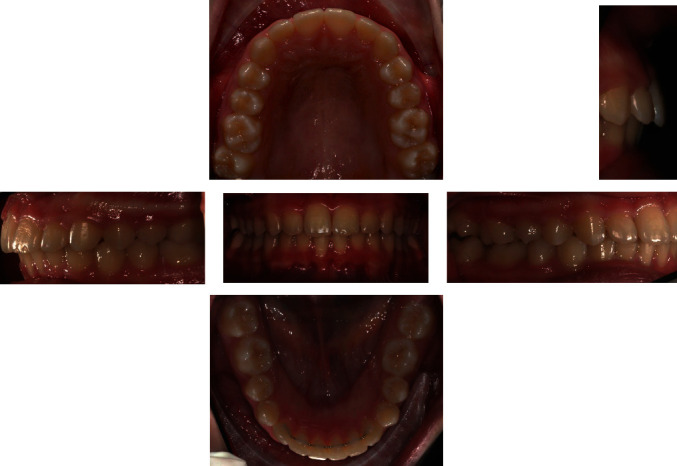
Final intraoral photograph.

**Figure 17:**
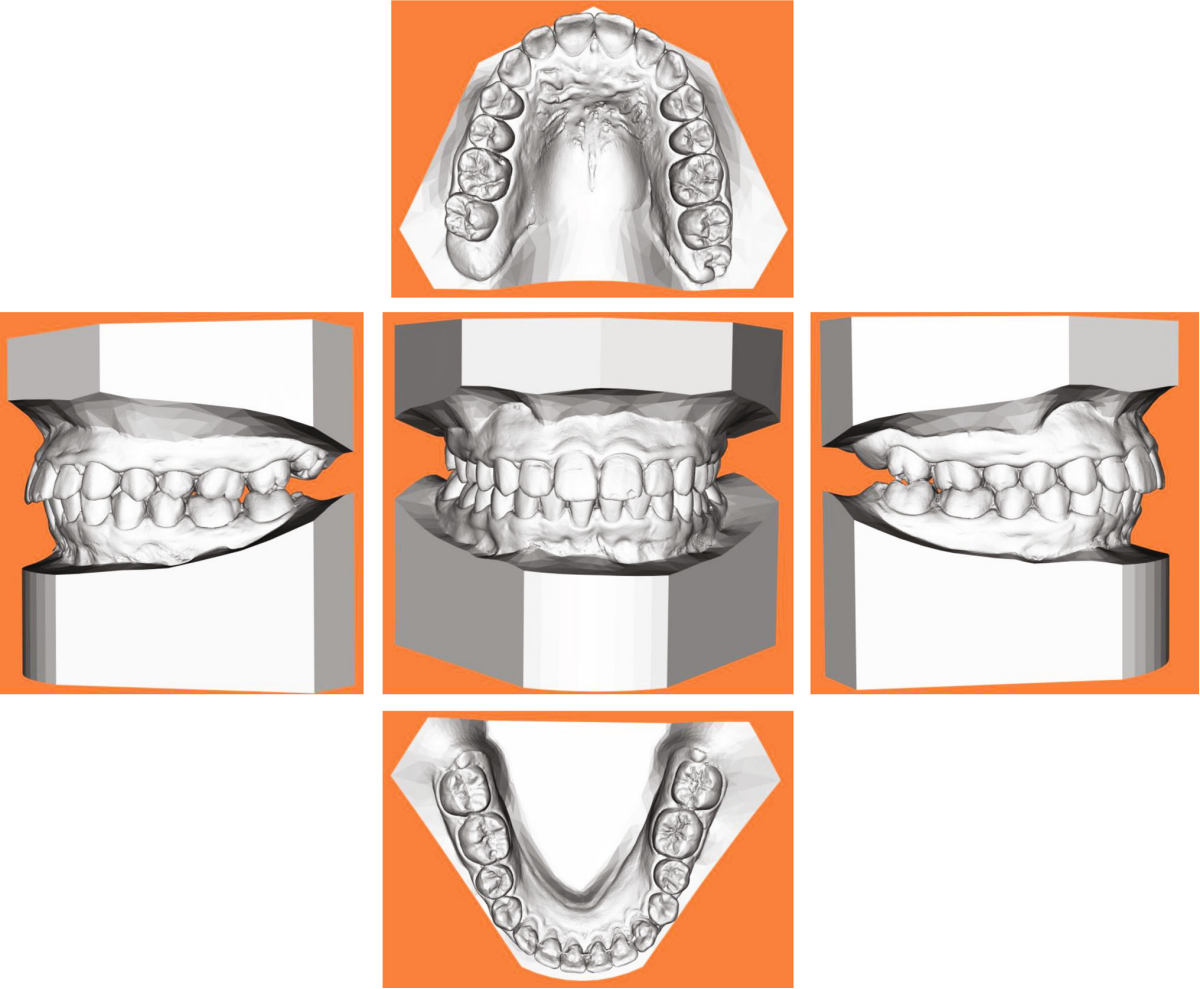
Final digital model.

At the end of the treatment, after a further year of orthodontic therapy with multibrackets, the patient presents a clear improvement in the profile with an evident in mandibular growth (Figures 13–15);from the intraoral photograph and from the mould (Figures 16 and 17), the achievement of a perfect canine and molar Class I is evident.

**Figure 18:**
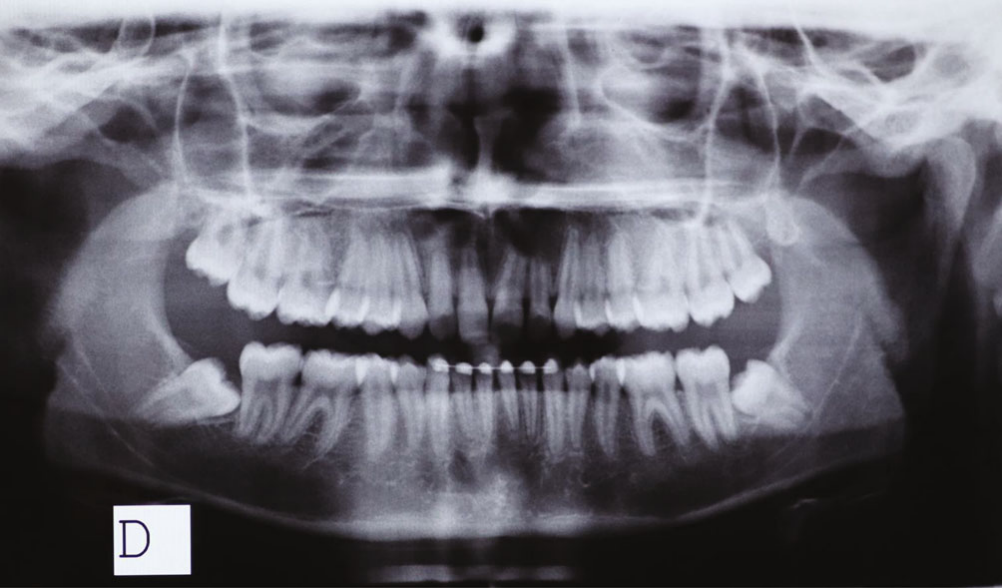
Final panoramic.

The panoramic X-ray shows a good radicular parallelism (Figure 18).

**Figure 19:**
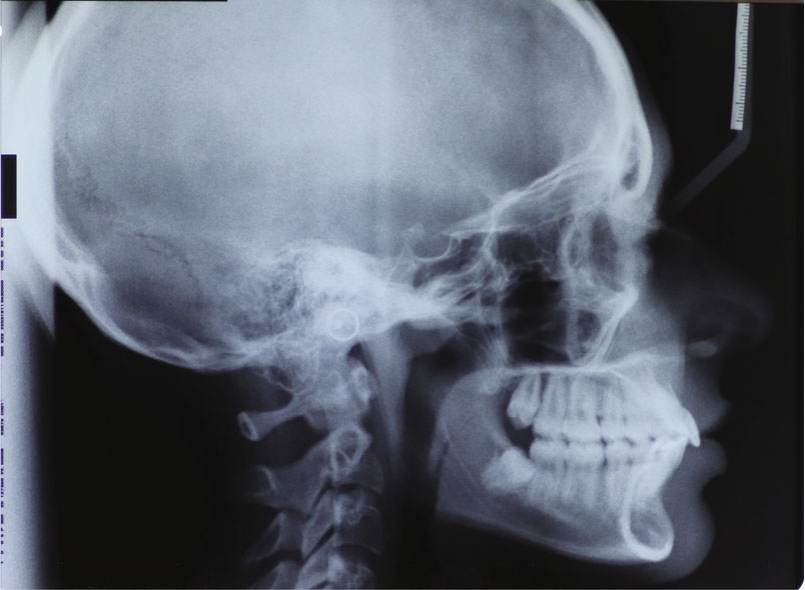
Final teleradiography.

The cephalometry at the end of the treatment [Figure 19; Tables 5 and 6] shows the following: according to Ebo values it is possible to register a stabilisation of the dental–skeletal Class I which goes from 4.4 to 3.6°, but above all, it can be noted that the position of the lower incisors compared with the value of cephalometry found after Herbst treatment (−1/Go-Gn pre/−1/Go-Gn post = +0.3°) remains unvaried just as the values according to Pancherz cephalometry do as well.

**Figure 20:**
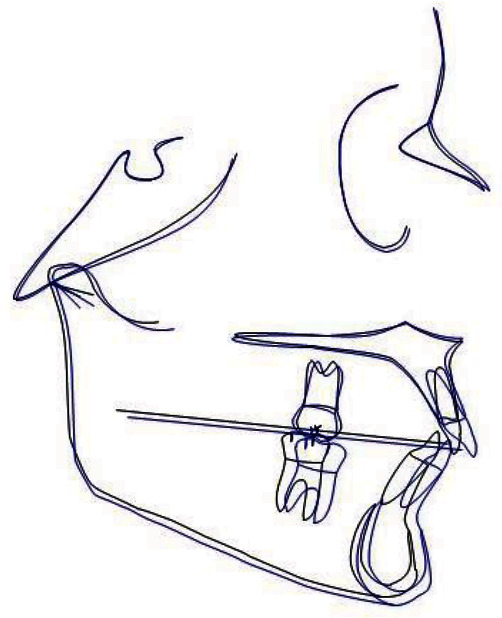
Cephalometric superimposition post-Herbst.

**Figure 21:**
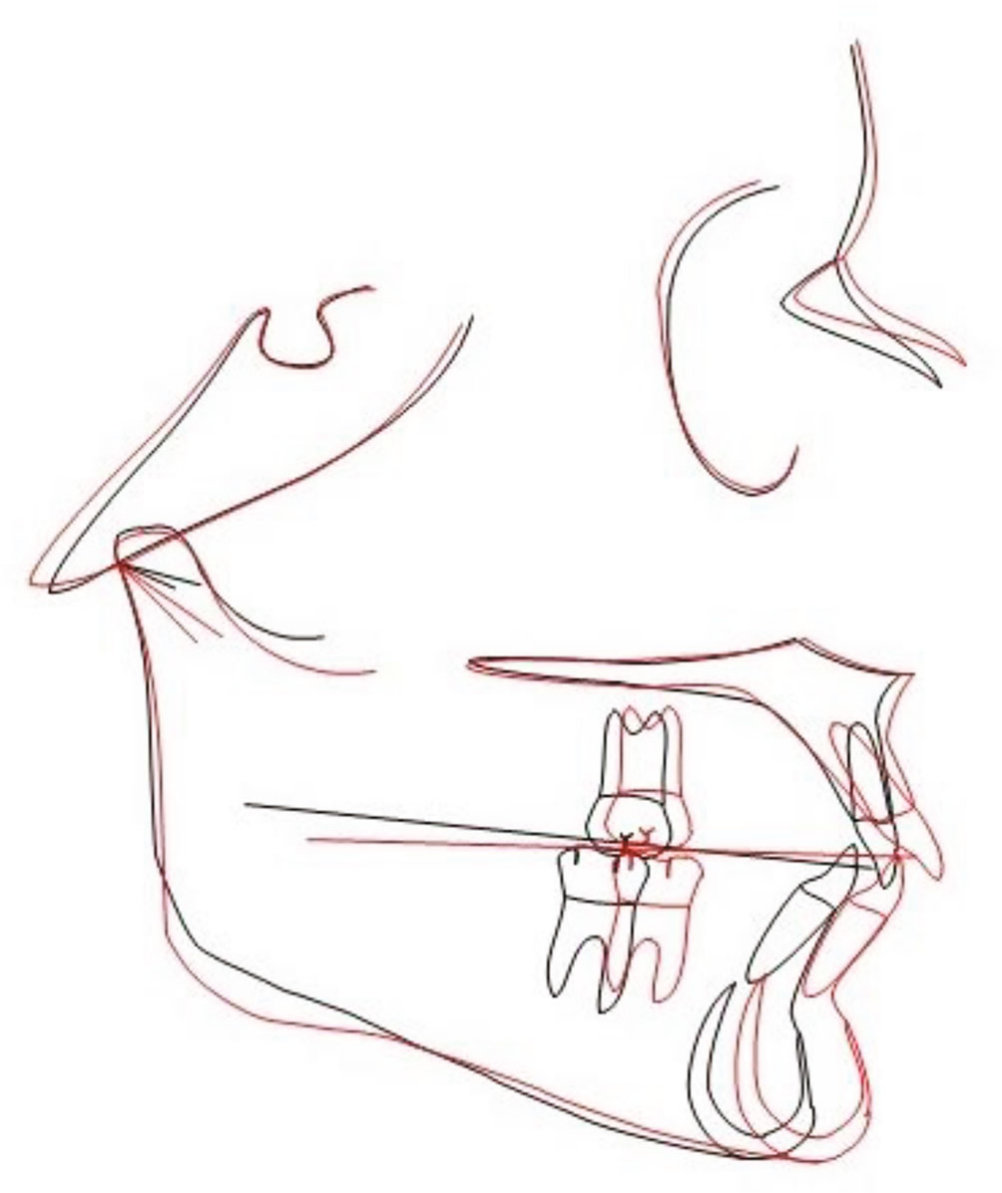
Cephalometric final superimposition.

The results of the treatment are easily assessed if the initial readings are superimposed on those at the time *T*1 (Figure 20) and at the end of the therapy (Figure 21).

## 8. Conclusions

The use of the Herbst device associated with vestibular TADs can be seen, in this case, to be very useful to avoid the proclination of the lower incisors, which remains stable even after orthodontic finishing.

## Figures and Tables

**Table 1:** Initial Ebo cephalometric values.

Sagittal skeletal relations		
Maxillary position S-N-A	86.7°	82° ± 3.5°
Mandibular position S-N-Pg	80.1°	80° ± 3.5°
Sagittal jaw relation A-N-Pg	6.6°	2° ± 2.5°
Vertical skeletal relations		
Maxillary inclination S-N/ANS-PNS	9.9°	8° ± 3.0°
Mandibular inclination S-N/Go-Gn	28°	33° ± 2.5°
Vertical jaw relation ANS-PNS/Go-Gn	18.1°	25° ± 6.0°
Dento-basal relations		
Maxillary incisor inclination 1-ANS-PNS	101.1°	110° ± 6.0°
Mandibular incisor inclination 1-Go-Gn	107.7°	94° ± 7.0°
Mandibular incisor compensation 1-A-Pg (mm)	−1.0	2° ± 2.0°
Dental relations		
Overjet (mm)	4.1	3.5° ± 2.5°
Overbite (mm)	4.4	2° ± 2.5°
Interincisal angle 1/1	133.1°	132° ± 6.0°

**Table 2:** Initial Pancherz cephalometric values.

Pancherz's analysis	
Sella-nasion line	62.4
OLp-U1 position	74.2
OLp-Prs	97.6
OLp-Ans	72.4
OLp-A	70.4
OLp-As	83.7
OLp-Sn	83.6
OLp-ULs	88.6
OLp-U6	38.8
OLp-L1	69.9
OLp-LLi	84.7
OLp-L6	36.3
OLp-B	65.6
OLp-Sub	75.8
OLp-Pg	69.4
OLp-Pos	81.0

**Table 3:** Post-Herbst Ebo cephalometric values.

Sagittal skeletal relations		
Maxillary position S-N-A	86°	82° ± 3.5°
Mandibular position S-N-Pg	81.6°	80° ± 3.5°
Sagittal jaw relation A-N-Pg	4.4°	2° ± 2.5°
Vertical skeletal relations		
Maxillary inclination S-N/ANS-PNS	8.0°	8° ± 3.0°
Mandibular inclination S-N/Go-Gn	26.4°	33° ± 2.5°
Vertical jaw relation ANS-PNS/Go-Gn	18.4°	25° ± 6.0°
Dento-basal relations		
Maxillary incisor inclination 1-ANS-PNS	116.8°	110° ± 6.0°
Mandibular incisor inclination 1-Go-Gn	107.7°	94° ± 7.0°
Mandibular incisor compensation 1-A-Pg (mm)	0	2° ± 2.0°
Dental relations		
Overjet (mm)	5.5	3.5° ± 2.5°
Overbite (mm)	3.0	2° ± 2.5°
Interincisal angle 1/1	117.1°	132° ± 6.0°

**Table 4:** Post-Herbst Pancherz cephalometrics values.

Pancherz's analysis	
Sella-nasion line	63.1
OLp-U1 position	78.6
OLp-Prs	98.5
OLp-Ans	72.5
OLp-A	71.4
OLp-As	83.1
OLp-Sn	84.6
OLp-ULs	85.9
OLp-U6	38.3
OLp-L1	73.0
OLp-LLi	86.5
OLp-L6	39.7
OLp-B	68.2
OLp-Sub	79.9
OLp-Pg	72.9
OLp-Pos	84.8

**Table 5:** Post-treatment Ebo cephalometric values.

Sagittal skeletal relations		
Maxillary position S-N-A	86.8°	82°±3.5°
Mandibular position S-N-Pg	83.2°	80°±3.5°
Sagittal jaw relation A-N-Pg	3.6°	2°±2.5°
Vertical skeletal relations		
Maxillary inclination S-N/ANS-PNS	9.5°	8°±3.0°
Mandibular inclination S-N/Go-Gn	23.1°	33°±2.5°
Vertical jaw relation ANS-PNS/Go-Gn	13.7°	25°±6.0°
Dento-basal relations		
Maxillary incisor inclination 1-ANS-PNS	124.9°	110°±6.0°
Mandibular incisor inclination 1-Go-Gn	108°	94°±7.0°
Mandibular incisor compensation 1-A-Pg (mm)	1.1	2 ± 2.0
Dental relations		
Overjet (mm)	4.6	3.5 ± 2.5
Overbite (mm)	2.8	2 ± 2.5
Interincisal angle 1/1	113.5°	132°±6.0°

**Table 6:** Post-treatment Pancherz cephalometrics values.

Pancherz's analysis	
Sella-nasion line	63.2
OLp-U1 position	78.9
OLp-Prs	100.8
OLp-Ans	73.4
OLp-A	71.0
OLp-As	84.6
OLp-Sn	85.4
OLp-ULs	87.3
OLp-U6	39.9
OLp-L1	74.0
OLp-LLi	87.8
OLp-L6	41.3
OLp-B	68.5
OLp-Sub	80.8
OLp-Pg	73.6
OLp-Pos	84.2

## Data Availability

Data supporting this research article are available from the corresponding author or first author on reasonable request.
